# MDD-carb: a combinatorial model for the identification of protein carbonylation sites with substrate motifs

**DOI:** 10.1186/s12918-017-0511-4

**Published:** 2017-12-21

**Authors:** Hui-Ju Kao, Shun-Long Weng, Kai-Yao Huang, Fergie Joanda Kaunang, Justin Bo-Kai Hsu, Chien-Hsun Huang, Tzong-Yi Lee

**Affiliations:** 1 Department of Computer Science and Engineering, Yuan Ze University, Taoyuan, city, 320 Taiwan; 2 Department of Medicine, Mackay Medical College, New Taipei City, 252 Taiwan; 3 Department of Obstetrics and Gynecology, Hsinchu Mackay Memorial Hospital, Hsinchu, city, 300 Taiwan; 4 Mackay Junior College of Medicine, Nursing and Management, Taipei, city, 112 Taiwan; 5 Department of Medical Research, Hsinchu Mackay Memorial Hospital, Hsinchu, city, 300 Taiwan; 6 Department of Medical Research, Taipei Medical University Hospital, Taipei, city, 110 Taiwan; 7 Tao-Yuan Hospital, Ministry of Health & Welfare, Taoyuan, 320 Taiwan; 8 Innovation Center for Big Data and Digital Convergence, Yuan Ze University, Taoyuan, 320 Taiwan

**Keywords:** Reactive oxygen species (ROS), Protein carbonylation, Substrate motifs, Profile hidden Markov model, Maximal dependence decomposition

## Abstract

**Background:**

Carbonylation, which takes place through oxidation of reactive oxygen species (ROS) on specific residues, is an irreversibly oxidative modification of proteins. It has been reported that the carbonylation is related to a number of metabolic or aging diseases including diabetes, chronic lung disease, Parkinson’s disease, and Alzheimer’s disease. Due to the lack of computational methods dedicated to exploring motif signatures of protein carbonylation sites, we were motivated to exploit an iterative statistical method to characterize and identify carbonylated sites with motif signatures.

**Results:**

By manually curating experimental data from research articles, we obtained 332, 144, 135, and 140 verified substrate sites for K (lysine), R (arginine), T (threonine), and P (proline) residues, respectively, from 241 carbonylated proteins. In order to examine the informative attributes for classifying between carbonylated and non-carbonylated sites, multifarious features including composition of twenty amino acids (AAC), composition of amino acid pairs (AAPC), position-specific scoring matrix (PSSM), and positional weighted matrix (PWM) were investigated in this study. Additionally, in an attempt to explore the motif signatures of carbonylation sites, an iterative statistical method was adopted to detect statistically significant dependencies of amino acid compositions between specific positions around substrate sites. Profile hidden Markov model (HMM) was then utilized to train a predictive model from each motif signature. Moreover, based on the method of support vector machine (SVM), we adopted it to construct an integrative model by combining the values of bit scores obtained from profile HMMs. The combinatorial model could provide an enhanced performance with evenly predictive sensitivity and specificity in the evaluation of cross-validation and independent testing.

**Conclusion:**

This study provides a new scheme for exploring potential motif signatures at substrate sites of protein carbonylation. The usefulness of the revealed motifs in the identification of carbonylated sites is demonstrated by their effective performance in cross-validation and independent testing. Finally, these substrate motifs were adopted to build an available online resource (MDD-Carb, http://csb.cse.yzu.edu.tw/MDDCarb/) and are also anticipated to facilitate the study of large-scale carbonylated proteomes.

**Electronic supplementary material:**

The online version of this article (10.1186/s12918-017-0511-4) contains supplementary material, which is available to authorized users.

## Background

Post-translational modifications (PTMs) are chemical modifications that take a significant part in various biological processes including transcriptional regulation, cell differentiation, apoptosis, signaling and metabolic pathways, protein activity, and protein-protein interactions [1, 2]. In most types of PTMs, enzymes are typically responsible for the attachment and removal of chemical groups on specific residue. Well-known examples are protein kinases that carry out phosphorylation of proteins in signaling pathways and phosphatases that carry out dephosphorylation [3]. However, several types of PTMs were reported that occur in a non-catalyzed manner, and are often influenced out by amino acid composition, structural environment, and physicochemical properties of proteins. These kinds of PTMs are known as non-enzymatic protein modifications, such as oxidation, *S*-nitrosylation, glutathionylation, carbonylation, isomerization, sulfenylation, deamidation, and glycation [4, 5]. Reactive Oxygen Species (ROS) play crucial roles in signaling networks as well as in the resistance of violating pathogens [6]. Oxidative stress occurs due to the abundance of ROS and the carbonylation of proteins is an irreversible PTM that has been regarded as a biomarker for oxidative stress based on its relative stability and ease of quantification [7, 8].

There are at least three mechanisms by which protein carbonylation occurs. The first one is direct oxidation by ROS on K (lysine), R (arginine), T (threonine), or P (proline) residues involving carbonyl derivatives of 2-pyrrolidone from proline, α-aminoadipic semialdehyde from lysine, glutamic semialdehyde from arginine and proline, as well as 2-amino-3-ketobutyric acid from threonine [6, 8, 9]. Previous studies has reported that the carbonylation is related to a number of metabolic or aging diseases including diabetes, chronic lung disease, Parkinson’s disease, and Alzheimer’s disease [5–7]. Because of the biological importance of protein carbonylation, mass spectrometry (MS)-based proteomics are widely employed to detect large-scale carbonylated peptides [10, 11]. However, the MS-based method for the identification of site-specific carbonylated peptides is labor-intensive and time-consuming. Therefore, several in silico approaches have been proposed for the prediction of carbonylated residues based on protein sequences. Additional file 1: Table S1 shows that, in 2014, Lv et al. developed a web tool, namely CarsPred, for identifying the carbonylation sites in human proteins using WSVM [12]. In 2016, Jia et al. developed a predictor called iCar-PseCp by incorporating sequence-coupled information into the general pseudo-amino acid composition, and balancing out skewed training datasets by Monte Carlo sampling to expand positive subsets [13]. This year, Weng et al. created an automatic scheme for providing a full study of substrate site preference in protein carbonylation [14]. Recently, a new approach named predCar-Site was designed to predict protein carbonylation sites by (1) incorporating sequence-coupled information into the general pseudo-amino acid composition, (2) balancing the effect of skewed training datasets by the Different Error Costs method, and (3) constructing a predictor using a support vector machine as a classifier [15]. The predCar-Site predictor could yield an average AUC (area under curve) score of 0.9959, 0.9999, 1, and 0.9997 for predictions in carbonylated K, P, R, and T, respectively.

The aim of this study is to characterize potential substrate motifs with an attempt to identify carbonylation sites. Herein, a variety of sequential attributes such as composition of amino acid (AAC), composition of amino acid pairs (AAPC), amino acid sequence (AA), positional weighted matrix (PWM), BLOSUM62 (B62), and position-specific scoring matrix (PSSM) were examined the ability to discriminate between carbonylation and non-carbonylation sites. Moreover, maximal dependence decomposition (MDD) [16], an iteratively statistical method, was employed to recognize motif patterns of carbonylation sites. MDD provides the possibility for a large group of aligned sequences to be partitioned into subgroups that contain consensus motifs based on the most remarkable dependencies of amino acid composition between positions around carbonylated sites. Each subgroup is then built as a predictive model with a corresponding MDD-identified motif using a profile hidden Markov model (HMM). Then, the support vector machine (SVM) is applied to build a combinatorial model by integrating the values of bit scores obtained from profile HMMs.

## Methods

### Collection and preprocessing of training dataset

The experimentally verified carbonylation peptides used in this study were obtained from dbPTM [1, 17, 18], which is a public PTM database created by manually curating experimental data from literature and systematically collecting PTM information from public domains. The collected dataset, which implicates full-length carbonylated protein sequences as well as K, R, T, and P carbonylated positions in mammalian proteins, is regarded as a training dataset. In total, there are 241 non-redundant carbonylated proteins containing 332, 144, 135, and 140 carbonylated sites in K, R, T, and P residues, respectively. As described in previous studies [19–24], the carbonylation sites were used as the positive training dataset, while the non-carbonylated K, R, T and P residues were used as the negative training dataset. As a typical study in computation prediction of PTM sites, an effective window size should be determined by using a sequence fragment having a window size of 2*n* + 1 amino acids and centering on carbonylated residues. The window length usually varies from 3 (*n* = 1) to 31 (*n* = 15) [25]. Based on the overall evaluation of various window lengths in a previous investigation [14], in this study, a 21-mer window length (*n* = 10) was chosen to extract the sequence fragments for the positive and negative training datasets.

In order to prevent overestimation of the performance of our predictive model, homologous sequences were eliminated from the training datasets. Employing the software package of CD-HIT [26] with a threshold of 50% sequence similarity, excessively similar sequences were removed from both the positive and negative datasets; this was done to remove any negative sequences that were similar to positive sequences. The final positive training dataset consisted of 256 carbonylated sequences for the K residue, 115 for R, 109 for T, and 109 sequences for P. However, the amount of negative samples was excessively large compared to the amount of positive samples. Thus, to avoid an unreasonably imbalanced classification between positive and negative instances, the numbers of sequences in the negative dataset were set to twice the size of the numbers in the positive dataset (2:1 ratio); random selection of negative samples resulted in 512 K, 230 R, 218 T, and 218 P non-carbonylated peptides in the negative training dataset (Table 1). To avoid skewing the results, the process of random sampling of the negative dataset was repeated 30 times to obtain an average performance for cross-validation.

**Table 1:** Number of positive and negative training sequences on K, R, T, and P residues

Residue	Number of carbonylated proteins	Dataset	Number of sequences	TOTAL
K	162	Positive	256	768
		Negative	512	
R	96	Positive	115	345
		Negative	230	
T	85	Positive	109	327
		Negative	218	
P	82	Positive	109	327
		Negative	218	

### Feature extraction and encoding

This work focused on the analysis of sequence-based characteristics around experimentally confirmed carbonylation sites. A 21-mer window length centering on carbonylated sites was adopted to extract fragmented sequences for the training datasets. There are 21 types of amino acids used in feature encoding, consisting of 20 native amino acids and 1 dummy amino acid (represented by a hyphen (−)). Amino acid composition (AAC) is the most usual sequence feature calculating the occurring frequency of twenty amino acids within a given sequence fragment. In this study, the sum of the *k* vectors {*x*_*i*_, *i* = 1, ..., *k*} was representing *k* fragmented sequences in the training dataset, in which positive and negative datasets are labeled with +1 and −1, respectively. Given a sequence fragment *k*, *f*_*k*_ *(n)* represents the number of occurrences of the 20 native amino acids, where *n* stands for 20 types of amino acid. Hence, the composition of twenty amino acids *P*_*k*_ *(n)* is computed as follows [27]:

1 $$ {P}_k\ (n)=\frac{f_k(n)}{\sum \limits_{n=1}^{20}{f}_k(n)}\kern1em n=1,2,\dots, 20 $$

The AAC vector of a sequence fragment *x*_*k*_ is then defined as

2 $$ {x}_k=\left[{P}_k(1),{P}_k(2),\dots, {P}_k(20)\right] $$

To encode the composition of the twenty amino acids around the carbonylation sites, the 20-dimensional vector *x*_*k*_ included 20 elements specifying the frequencies of twenty amino acids normalized by the total number of amino acids in a fragmented sequence. The composition of amino acid pairs (AAPC) [28], which is similar to the AAC feature, transforms a sequence fragment into a 400-dimensional vector, which includes 400 elements specifying the numbers of occurrences of 400 amino acid pairs divided by the total number of amino acid pairs in a fragmented sequence. Additionally, an orthogonal binary coding method was used to transform each amino acid into a numeric vector. For example, Alanine (A) can be encoded as “10,000,000,000,000,000,000,” Cysteine (C) can be encoded as “01000000000000000000,” Aspartic acid (D) can be encoded as “0010000000000000000,” and so on. Given a fragmented sequence with a window size of 2*n* + 1, the number of dimensions in an orthogonal binary vector that represents the upstream and downstream amino acids around the central position (carbonylated site) was (2*n* + 1) × 20.

According to the theory of structural conservation, a number of amino acids might be mutated without changing the structural conformation of a protein [29]. Hence, two proteins may have similar structures but different compositions of amino acids. A position Specific Scoring Matrix (PSSM) was used to generate a profile of distantly-related residues from a cluster of sequences that was formerly aligned in structural resemblance [30]. PSSM profiles have been extensively utilized in the prediction of protein secondary structure, subcellular localization, and PTM substrate sites [20, 22, 29, 31–37]. By running a PSI-BLAST [38] against the database of non-homologous carbonylated sequences, a PSSM profile was generated with a matrix of (2*n* + 1) × 20 elements with the carbonylated site located in a central position. Rows with the same types of amino acids in the PSSM matrix were summed to obtain a matrix of 20 × 20 elements. Lastly, each element of the 20 × 20 matrix was divided by the window length 2*n* + 1 (*n* = 10) and normalized using the formula: $\frac{1}{1+{e}^{-x}}$.

As described in the coding method of SulfoSite [39], the positional weighted matrix (PWM) of amino acids surrounding the carbonylated site was determined by calculating the relative frequency of 20 amino acids at a specific position. After the construction of the PWM from the positive training dataset, each sequence fragment was transformed into a numeric vector with *(*2*n* + 1) × *w* elements, where 2*n* + 1 denoted the window size while *w* represented the frequencies of the 20 amino acids. Additionally, the BLOSUM62 (amino acid substitution matrix) was generated based on the alignments of peptide sequences having less than 62% sequence identity. Each sequence fragment was transformed into a numeric vector according to the substitution scores of twenty amino acids from BLOSUM62.

### Detection of substrate motifs by maximal dependence decomposition

**Fig. 1:**
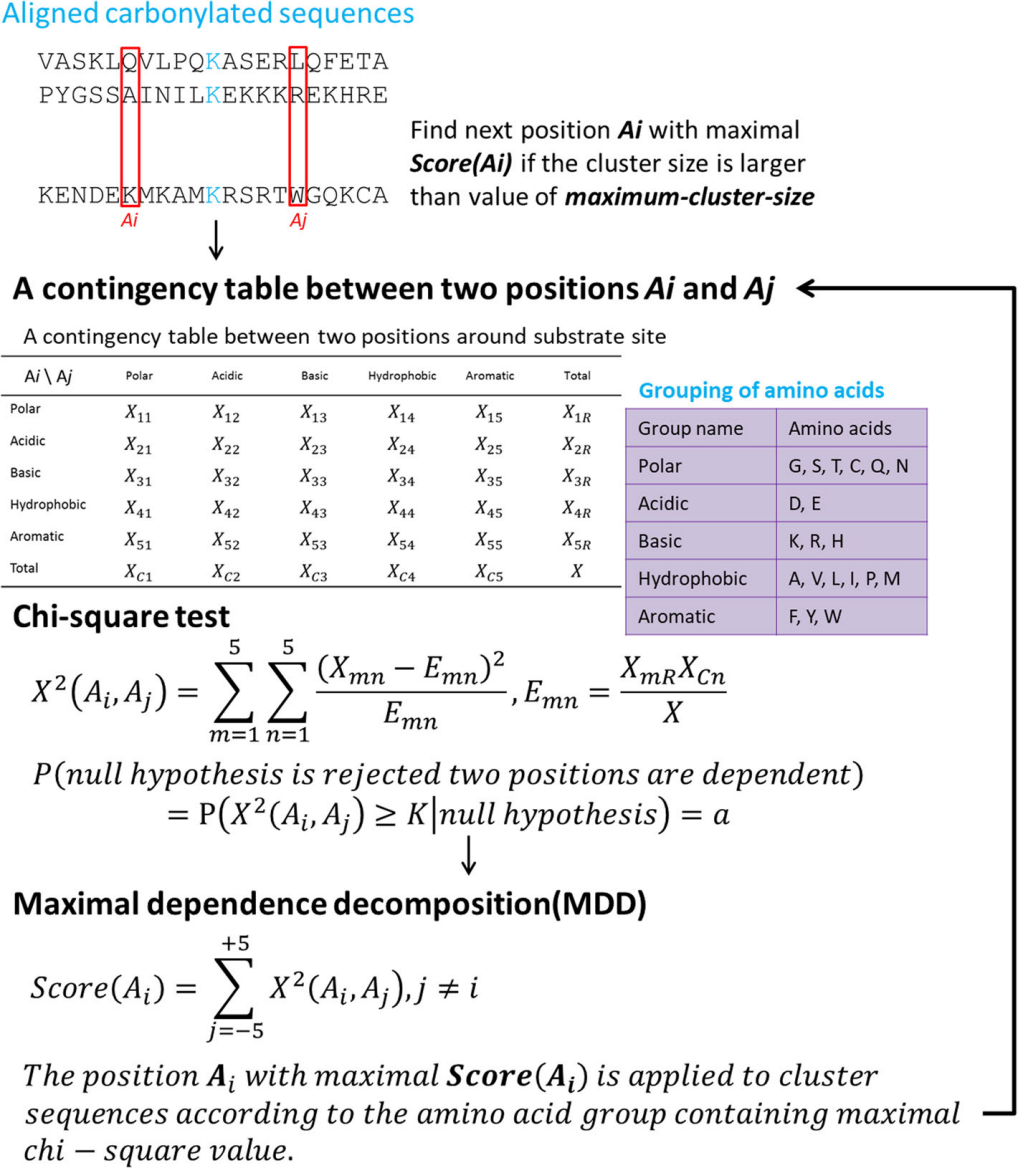
Analytical flowchart of maximal dependence decomposition

Based on the amino acid sequences, the motif signatures of the substrate sites were explored around the carbonylated residues. The positive training dataset (carbonylated sequence fragments) was used to investigate the substrate motifs based on maximal dependence decomposition (MDD) [16]. Due to the difficulty of observing the conserved motifs from a large-scale sequence dataset, MDD has been utilized to cluster a group of aligned phosphorylated peptides into subgroups that show statistically significant motifs [20]. Previous studies [31, 35, 40–42] have demonstrated the effectiveness of the clustering of modified sequences into subgroups prior to the construction of predictive models. For this investigation, MDD was applied using public software, MDDLogo [31], to cluster all the sequence fragments of the positive training dataset. The kernel of MDDLogo applied the chi-squared test to iteratively evaluate the correlation between the occurrence of amino acids between two positions, Ai and Aj, neighboring the carbonylated site. To avoid a higher degree of freedom in the chi-squared test, the 20 types of amino acids were categorized into five groups according to biochemical properties, including polarity, acidity, basicity, hydrophobicity, and aromaticity, as shown in Fig. 1. To evaluate the dependence of amino acid occurrence between two positions (*A*_*i*_ and *A*_*j*_) surrounding the carbonylated sites, a chi-squared test *x* ^2^(*Ai*, *Aj*) was performed as follows:

3 $$ {\chi}^2\left({\mathrm{A}}_{\mathrm{i}},{\mathrm{A}}_{\mathrm{j}}\right)=\sum \limits_{m=1}^5\sum \limits_{n=1}^5\frac{{\left({X}_{mn}-{E}_{mn}\right)}^2}{E_{mn}} $$

The number of sequences at the position *A*_*i*_ of the group *m* and position *A*_*j*_ of group *n* are represented by *X*_*mn*_ for each pair (*A*_*i*_ and *A*_*j*_) and *i* ≠ *j*. *X* represents the total number of sequences and *E*_*mn*_ was projected as $\frac{X_{mR}\cdot {X}_{Cn}}{X}$, where *X*_*mR*_ = *X*_*m1*_+ … + *X*_*m5*_, *X*_*Cn*_ = *X*_*1n*_ + … + *X*_*5n*_. To determine the value of the chi-squared test, a contingency table describing the co-occurrence of amino acids between *A*_*i*_ and *A*_*j*_ was provided. Given *A*_*i*_ and *A*_*j*_, if the value of the chi-squared test was larger than 34.3, based on degrees of freedom =(5 − 1) × (5 − 1) and *p-*value *≤*0.005, the null hypothesis was rejected because the two positions were dependent. The process was then repeated as described by Burge and Karlin [43]. MDDLogo provided a tree-like visualization for the hierarchical clustering of the positive training dataset. Since MDDLogo was applied on the positive training dataset, the parameter of maximum-cluster-size was set in order to terminate the MDD clustering process. If the size of a subgroup was less than the value of maximum-cluster-size, the subgroup was not divided any further and the process of hierarchical clustering was terminated until the sizes of all subgroups were smaller than the value of maximum-cluster-size.

### Construction of predictive models

A support vector machine (SVM) [44] is an advanced machine learning method for pattern recognition and data classification. Based on the binary classification between the positive and negative samples in this study, an SVM can transform all samples into a vector space of higher dimension by using different kernel functions. A hyperplane is then determined for discriminating between the positive and negative samples with maximal margin and minimal error. Various sequence-based features are encoded as numeric vectors for input in the SVM. Herein, a popular SVM library, LIBSVM [45], was installed in our computing server in order to efficiently build a predictive model for each feature. LIBSVM provides four kernel functions, namely a linear function, polynomial function, radial basis function (RBF), and sigmoid function, for the transformation of sample space. As described in a number of previous works [3, 22, 24, 46, 47], the RBF is a reasonably best choice for a kernel function when training an SVM classifier. The RBF function is defined as *K*(*S*_*i*_, *S*_*j*_) = exp(−*γ*‖*S*_*i*_ − *S*_*j*_‖^2^). Two supporting parameters, gamma (*r*) and cost (*c*), are used to enhance the predictive power of the SVM. The RBF kernel is typically optimized by the gamma parameter, and the softness of hyperplane is modulated by the cost parameter. A Python program (grid.py) provided by LIBSVM was used to optimize gamma and cost and obtain better predictive accuracy.

In addition to the RBF-based SVM, a profile hidden Markov model (HMM) was also used to generate the predictive model, especially for the MDD-identified motif signatures. A profile HMM can determine the probability distribution of 20 amino acids against large-scale sequence data and can detect distant relationships between two positions surrounding the carbonylation sites [48]. In this study, the software package HMMER version 2.3.2 [48] was adopted to train and calibrate profile HMMs based on the positive training dataset. Furthermore, the profile HMM can be used to search the putative carbonylated sites on a protein sequence. In an attempt to capture the characteristics of each MDD-identified motif, each of the MDDLogo-clustered subgroups was regarded as a training dataset for training a profile HMM. When searching the hits of a profile HMM, HMMER returns a bit score and an expectation value (E-value). Given an input sequence, a positive prediction is defined as the HMMER bit score greater than the threshold parameter. If the bit score threshold is defined as a lower value, the predicted result will induce a higher sensitivity (true positive prediction rate); oppositely, increasing the bit score threshold favors a higher specificity (true negative prediction rate). Hence, the threshold value should be optimized for achieving a better performance with balanced sensitivity and specificity. After the construction of a profile HMM for each MDDLogo-clustered subgroup (first layer) and for each carbonylated residue type, all profile HMMs were integrated into a combinatorial model using the SVM. As presented in Additional file 2: Figure S1, the values of bit scores obtained from the profile HMMs were used to form a numeric vector of bit scores for constructing an SVM classifier in the second layer.

### Performance evaluation

#### Five-fold cross-validation

In this work, the performances of the predictive models trained with various features were evaluated based on five-fold cross-validation. Firstly, all sequences of training dataset were randomly split into five approximately equal-sized subgroups. Among the five subgroups, one was used as the validation data and the remaining four subgroups were used as the training data. Then, the process was executed five times where each subgroup should be regarded as the validation set in turn. The predicted results of five validation sets were then combined into a single performance. Finally, the performance of the predictive models was determined based on the following metrics:

4 $$ {S}_n=\frac{TP}{TP+ FN} $$

5 $$ {S}_p=\frac{TN}{TN+ FP} $$

6 $$ Acc=\frac{TP+ TN}{TP+ TN+ FP+ FN} $$

7 $$ MCC=\frac{\left( TP\times TN\right)-\left( FP\times FN\right)}{\sqrt{\left( TP+ FN\right)\times \left( TN+ FP\right)\times \left( TP+ FP\right)\times \left( TN\times FN\right)}} $$

where TP, TN, FP, and FN represent the numbers of true positives, true negatives, false positives, and false negatives, respectively. The *Sn* (sensitivity) and *Sp* (specificity) indicate the accurate prediction ratios of positive (carbonylation) and negative (non-carbonylation) results, respectively. The *Acc* (accuracy) denotes the ratio of correct prediction of true positives and true negatives. In unbalanced positive and negative datasets, the Matthews correlations coefficient (*MCC*) is a convenient benchmark for the correlation between the observed and predicted classifications of the positive and negative samples. The MCC value ranges from −1 to +1, where the value of +1 represents a perfectly correct classification, while the values 0 and −1 represent a random prediction and perfectly wrong classification, respectively. Furthermore, the ROC (Receiver Operating Characteristic) curve of various models is used for the comparison of AUC (area under the curve of ROC) values.

### Independent testing

In order to compare the proposed method with other prediction tools, an independent testing dataset, which is truly blind to the training dataset, was constructed by manually curating eight research articles [49–56], which extracted 132 K, 102 R, 82 T, and 104 P carbonylation sites on 80, 71, 62, and 71 carbonylated proteins, respectively, from multiple species. After the removal of homologous sequences by using the CD-HIT program, the final testing dataset comprised 85, 72, 63, and 82 carbonylation sites on K, R, T, and P, respectively (Table 2). Additionally, the negative dataset for independent testing was composed of 170 K, 144 R, 126 T, and 164 P non-carbonylation sites. An effective classification between positive and negative testing datasets would indicate a reliable and stable performance in the prediction of protein carbonylation sites.

**Table 2:** Number of positive and negative testing sequences on K, R, T, and P residues

Residue	Number of carbonylated proteins	Dataset	Number of sequences	TOTAL
K	80	Positive	85	255
		Negative	170	
R	71	Positive	72	216
		Negative	144	
T	62	Positive	63	189
		Negative	126	
P	71	Positive	82	246
		Negative	164	

## Results and discussion

### Investigation of amino acid composition at carbonylated sites

**Fig. 2:**
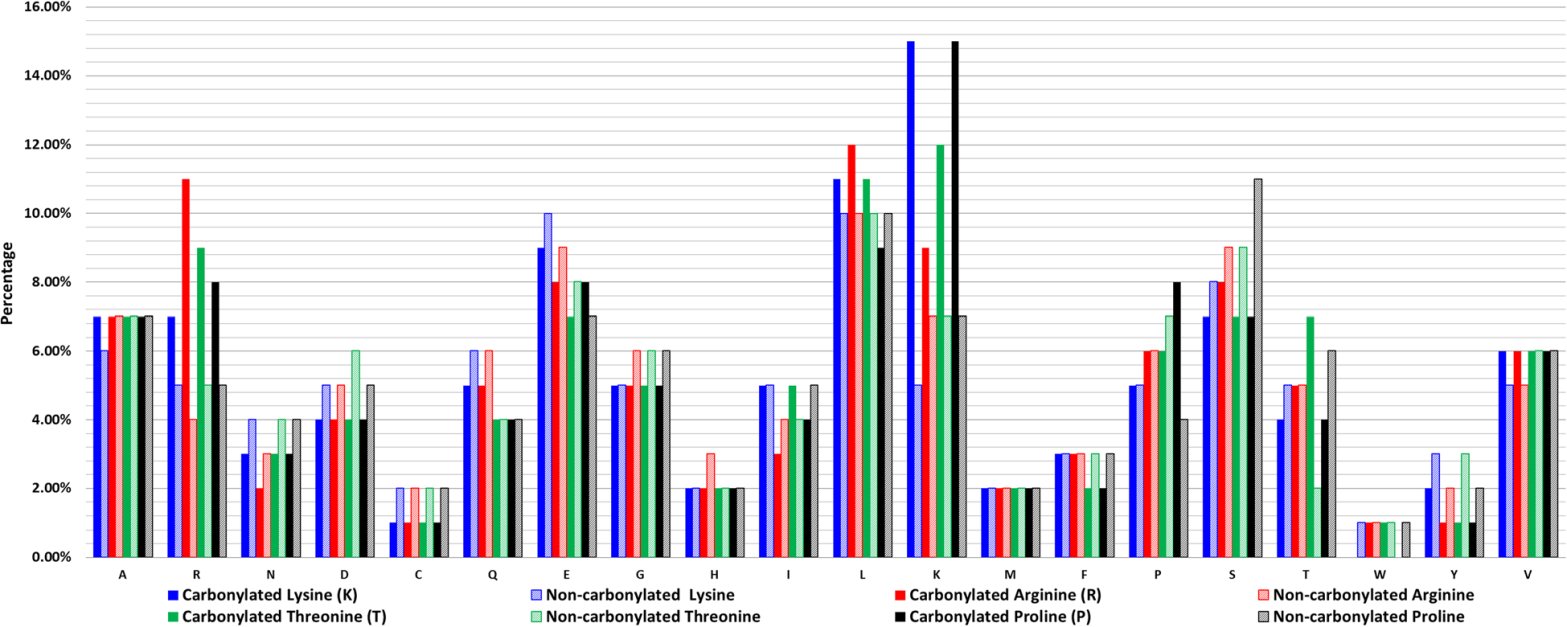
Amino acid composition of carbonylation and non-carbonylation sites on K, R, T and P residues

To study the composition of amino acids around carbonylated sites, a graphical representation was prepared by calculating the occurrence of each amino acid surrounding the carbonylation sites (the central amino acid, which is the carbonylation site, is excluded from the calculation) and divided by the length of the fragment excluded at the carbonylation site. This process was conducted for each carbonylation site (positive) and non-carbonylation site (negative). Figure 2 shows the comparisons of amino acid compositions in the positive and negative training datasets. We observed that the occurrence rates of K, R, T, and P residues in the carbonylation sites were higher than those in the non-carbonylation sites; K was significantly abundant in carbonylation sites. This investigation showed that a carbonylation site generally occurs within KRTP-abundant regions, which is consistent with findings reported by Nystrom et al. [57]. Additionally, we observed a dominant proportion of leucine (L); however, the reason for this is unknown and warrants further study. Additionally, in order to explore the position-specific composition of amino acids around carbonylation sites, frequency plots of the vicinities around carbonylated sites were graphically represented using WebLogo [58] and are provided in Additional file 3: Fig.ure S2. The frequency plots revealed that K and R residues are slightly enriched within the neighboring regions of carbonylation sites.

### Cross-validation evaluation of various features in carbonylation site prediction

In order to identify the most useful features in the classification of carbonylated and non-carbonylated sites, the SVM models trained with various features were evaluated based on five metrics including sensitivity (Sn), specificity (Sp), accuracy (Acc), Matthew’s correlation coefficient (MCC), and area under ROC curve (AUC). Based on the evaluation using five-fold cross-validation, the predictive performance of each sequence-based feature is presented in Table 3. In the prediction of K carbonylation sites, the SVM models trained with AAC and with PWM yield the best performance with an accuracy of 0.69, MCC value of 0.37, and AUC of 0.78. For the prediction of R carbonylation sites, the SVM model trained with PWM provided the best performance with a sensitivity of 0.71, specificity of 0.70, accuracy of 0.70, MCC value of 0.39, and AUC of 0.80 in discriminating between 115 carbonylated and 230 non-carbonylated R sites. In the classification between 109 carbonylated and 218 non-carbonylated T sites, the AAC model performed best with a sensitivity of 0.74, specificity of 0.70, accuracy of 0.72, MCC value of 0.41, and AUC of 0.82. For carbonylated P sites, the SVM model trained from PWM provided the best prediction with a sensitivity of 0.72, specificity of 0.73, accuracy of 0.73, MCC value of 0.42, and AUC of 0.82. Additionally, the SVM model trained with AAC is comparable to that trained with PWM in discriminating between 109 carbonylated and 218 non-carbonylated P sites. In short, the SVM models trained with AAC or with PWM provided the best performance in identifying carbonylation sites. Moreover, the comparison of ROC curves among the SVM models trained with various features for the identification of carbonylated K, R, T, and P sites are given in Additional file 4: Figure S3, Additional file 5: Figure S4, Additional file 6: Figure S5, and Additional file 7: Figure S6. In an attempt to detect distant relationships between positions around the carbonylation sites, a profile HMM was also used to generate a predictive model for identifying carbonylated sites. The comparison of ROC curves indicated that the profile HMMs could provide a comparable performance to the SVM models trained with AAC or with PWM.

**Table 3:** Five-fold cross-validation results of the SVM models trained with various features for discriminating between positive and negative training datasets

Residue	Training features	Sn	Sp	Acc	MCC	AUC
K	Amino acid composition (AAC)	0.70	0.69	0.69	0.37	0.78
	Amino acid pairs composition (AAPC)	0.66	0.65	0.65	0.29	0.71
	Amino acid sequence (AA)	0.68	0.64	0.65	0.23	0.67
	Positional weighted matrix (PWM)	0.74	0.67	0.69	0.37	0.78
	Position specific scoring matrix (PSSM)	0.63	0.61	0.62	0.16	0.61
	BLOSUM62 (B62)	0.63	0.60	0.61	0.15	0.59
R	Amino acid composition (AAC)	0.66	0.63	0.64	0.28	0.70
	Amino acid pairs composition (AAPC)	0.62	0.61	0.61	0.22	0.65
	Amino acid sequence (AA)	0.62	0.62	0.62	0.17	0.62
	Positional weighted matrix (PWM)	0.71	0.70	0.70	0.39	0.80
	Position specific scoring matrix (PSSM)	0.61	0.56	0.58	0.14	0.59
	BLOSUM62 (B62)	0.62	0.62	0.62	0.17	0.62
T	Amino acid composition (AAC)	0.74	0.70	0.72	0.41	0.82
	Amino acid pairs composition (AAPC)	0.69	0.68	0.69	0.35	0.75
	Amino acid sequence (AA)	0.63	0.62	0.62	0.18	0.63
	Positional weighted matrix (PWM)	0.69	0.67	0.68	0.32	0.73
	Position specific scoring matrix (PSSM)	0.65	0.65	0.65	0.29	0.70
	BLOSUM62 (B62)	0.58	0.50	0.53	0.08	0.53
P	Amino acid composition (AAC)	0.72	0.70	0.70	0.39	0.80
	Amino acid pairs composition (AAPC)	0.68	0.64	0.65	0.30	0.71
	Amino acid sequence (AA)	0.64	0.66	0.65	0.23	0.67
	Positional weighted matrix (PWM)	0.72	0.73	0.73	0.42	0.82
	Position specific scoring matrix (PSSM)	0.66	0.68	0.67	0.32	0.73
	BLOSUM62 (B62)	0.61	0.58	0.59	0.15	0.60

### MDDLogo-identified substrate motifs and their predictive performances

**Fig. 3:**
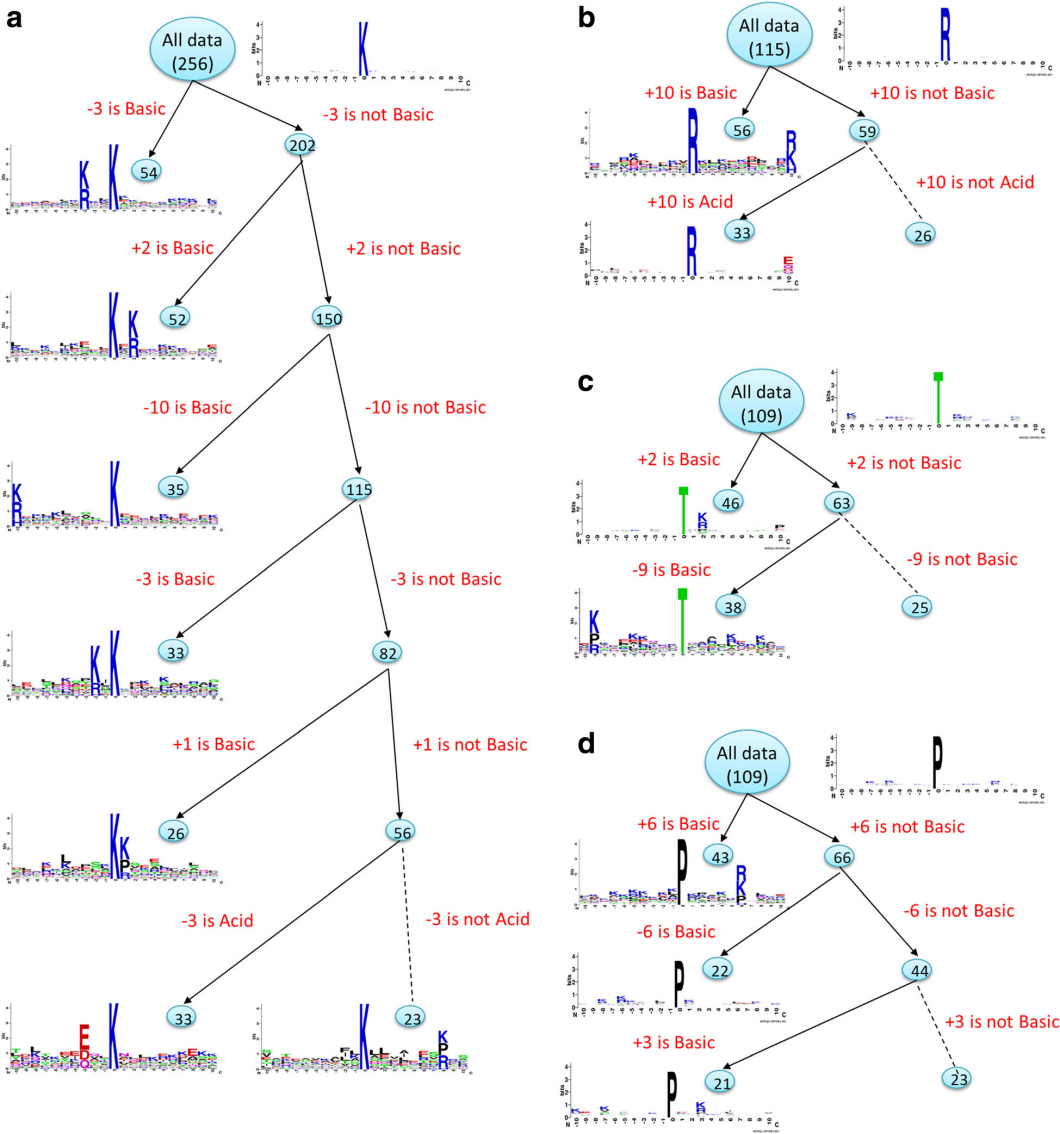
MDDLogo-identified substrate motifs of carbonylated (**a**) K, (**b**) R, (**c**) T, and (**d**) P sites

To identify the potential conserved motifs, we applied MDDLogo to cluster the positive training dataset into several subgroups which contain statistically significant dependencies of amino acid composition between specific positions of carbonylation sites. To specify whether the MDD-clustered subgroup contained potential conserved motifs, each subgroup was generated by WebLogo [58]. As shown in Fig. 3, of all the substrate motifs represented by each subgroup, we found that the substrate motifs were dominated by the positively charged amino acids (K, R, and H) and only two of the subgroups were detected based on the negatively charged amino acids (D, E). This finding shows that carbonylation is prone to occur in a basic environment. Additionally, these results demonstrated that the maximal dependent values of the basic group of amino acids were kept at position −3 for K carbonylation sites (Fig. 3a), position +10 for R carbonylation sites (Fig. 3b), position +2 for T carbonylation sites (Fig. 3c), and position +6 for P carbonylation sites (Fig. 3d). This MDD clustering process was repeatedly executed to hierarchically divide the positive datasets into tree-like subgroups whose data sizes were smaller than the value of maximum-cluster-size.

Among the MDDLogo-clustered subgroups of K carbonylation sites, subgroup CarbK_1, which had a conserved motif of K and R residues at position −3, yielded the best performance with a sensitivity of 0.81, specificity of 0.81, accuracy of 0.81, MCC value of 0.61, and AUC of 0.91 in discriminating between 54 carbonylated and 108 non-carbonylated K sites (Additional file 8: Table S2). In the prediction of K carbonylation sites, overall, the profile HMMs trained from the MDD-identified motif signatures provided better performances than those trained from all 256 carbonylated K sites without MDD clustering. For prediction of carbonylation sites on R residues, two subgroups containing statistically significant motifs with *p-values* less than 0.005 were detected by MDDLogo. Subgroup CarbR_1, which had a conserved motif of positively charged residues (R, K, and H) at position +10, provided the best performance with a sensitivity of 0.76, specificity of 0.74, accuracy of 0.75, MCC value of 0.47, and AUC of 0.85. For prediction of T carbonylation sites, the subgroup CarbT_2, possessing the motif of K/P/R at position −9, provided higher values for sensitivity (0.75), specificity (0.75), accuracy (0.75), MCC (0.48), and AUC (0.85) than the other subgroups. For carbonylated P sites, three substrate motifs were identified by MDDLogo. Of them, the subgroup CarbP_1, which contained a conserved R/K/P at position +6, achieved the best predictive performance. However, the subgroup CarbP_3 showed a slightly lower predictive performance than the model trained from all carbonylated P sites, which may have been caused by the small size of the positive training dataset. Overall, the profile HMMs trained from MDDLogo-clustered subgroups, which contain statistically significant motif signatures, presented enhanced performance compared to that of the models without MDD clustering.

### Performance evaluation by independent testing datasets

In the prediction of PTM substrate sites, it is possible to overestimate constructed models by overfitting to the training dataset. Thus, an independent testing dataset was employed to evaluate the real performance of the selected models with better MCC values. The testing results showed that, in K, R, T, and P carbonylation sites, the profile HMM trained from all positive training dataset yielded similar performance to the SVM models trained with AAC or with PWM. When using multiple profile HMMs trained from the MDDLogo-identified motifs, a higher sensitivity was obtained, accompanied by a lower specificity in the classification between positive and negative testing datasets on carbonylated K, R, T, and P sites. This investigation indicated that, after applying MDD clustering on positive training datasets, the multiple models typically induced higher true-positive predictions as well as higher false-positive predictions than did a single predictive model. To provide a reasonable integration of multiple profile HMMs, a combinatorial machine learning method was adopted, as described in a previous study [21]. This combinatorial model incorporated multiple profile HMMs into a single predictive model. Since each profile HMM was built from each of the MDDLogo-identified motifs, the LIBSVM was utilized to generate an integrative model (MDD-Carb) by combining the values of bit scores obtained from multiple profile HMMs. As presented in Table 4, the combinatorial model yielded the sensitivities of 0.80, 0.79, 0.79, and 0.77; specificities of 0.76, 0.73, 0.76, and 0.74; accuracies of 0.77, 0.75, 0.77, and 0.75; as well as MCC values of 0.53, 0.49, 0.53, and 0.49; for K, R, T, and P carbonylation sites, respectively. Although the combinatorial model performs at lower sensitivity than multiple profile HMMs, the overall best performance was obtained by incorporating multiple profile HMMs into a single SVM model.

**Table 4:** Comparison of independent testing results among various models in this work

Residue	Model	Sn	Sp	Acc	MCC	AUC
K	Single SVM trained with AAC	0.65	0.68	0.67	0.31	0.72
	Single SVM trained with PWM	0.67	0.68	0.68	0.33	0.73
	Single profile HMM trained from all data	0.69	0.68	0.69	0.35	0.74
	Multiple profile HMMs trained from MDDLogo-clustered subgroups	0.85	0.47	0.60	0.31	0.68
	Single SVM trained from multiple profile HMMs (MDD-Carb)	0.80	0.76	0.77	0.53	0.84
R	Single SVM trained with AAC	0.62	0.62	0.62	0.23	0.66
	Single SVM trained with PWM	0.65	0.65	0.65	0.29	0.70
	Single profile HMM trained from all data	0.68	0.66	0.67	0.33	0.72
	Multiple profile HMMs trained from MDDLogo-clustered subgroups	0.90	0.55	0.67	0.44	0.81
	Single SVM trained from multiple profile HMMs (MDD-Carb)	0.79	0.73	0.75	0.49	0.83
T	Single SVM trained with AAC	0.63	0.71	0.69	0.34	0.73
	Single SVM trained with PWM	0.67	0.71	0.70	0.36	0.74
	Single profile HMM trained from all data	0.67	0.71	0.70	0.36	0.74
	Multiple profile HMMs trained from MDDLogo-clustered subgroups	0.93	0.56	0.69	0.48	0.80
	Single SVM trained from multiple profile HMMs (MDD-Carb)	0.79	0.76	0.77	0.53	0.84
P	Single SVM trained with AAC	0.63	0.61	0.62	0.23	0.66
	Single SVM trained with PWM	0.69	0.67	0.68	0.34	0.74
	Single profile HMM trained from all data	0.69	0.67	0.68	0.34	0.74
	Multiple profile HMMs trained from MDDLogo-clustered subgroups	0.88	0.49	0.62	0.35	0.76
	Single SVM trained from multiple profile HMMs (MDD-Carb)	0.77	0.74	0.75	0.49	0.82

### Comparison with existing prediction tools

**Fig. 4:**
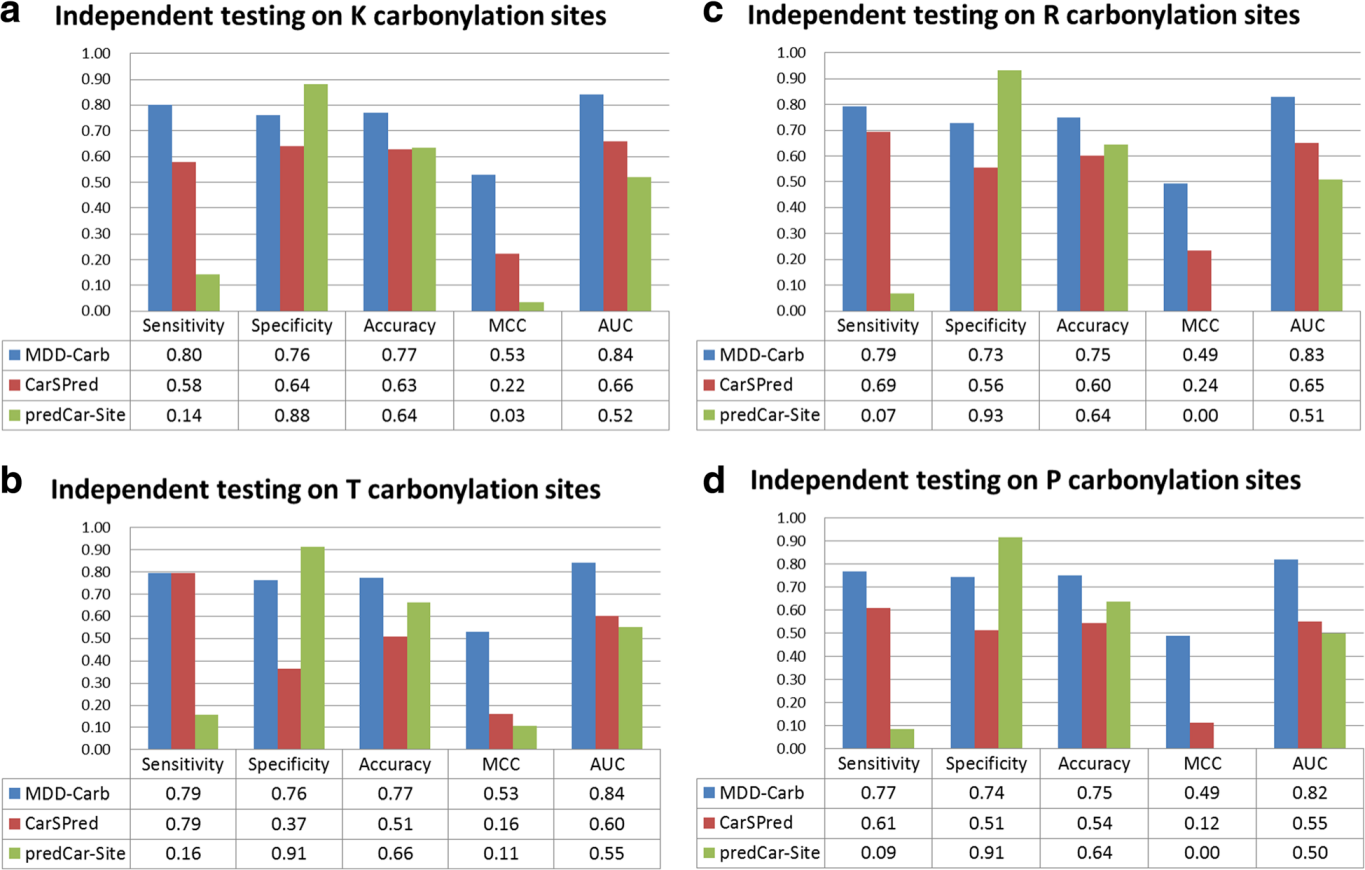
Comparison of independent testing results between MDD-Carb and two existing prediction tools. (**a**) Independent testing results on K carbonylation sites, (**b**) Independent testing results on  T carbonylation sites, (**c**) Independent testing results on R carbonylation sites, and (**d**) Independent testing results on P carbonylation sites

Considering the accessibility of previously published prediction tools, two online tools, CarSPred and predCar-Site, are available for the comparison of predictive performance based on independent testing datasets. Figure 4 showed that the predCar-Site (green bars) can yield the highest specificity values of 0.88, 0.93, 0.91, and 0.91 in the prediction of K, R, T, and P carbonylation sites, respectively. However, the high true-negative prediction of predCar-Site induces a very low sensitivity in the identification of positive testing datasets. Although the present method (MDD-Carb) could not provide better specificity comparing to predCar-Site, the results of independent testing demonstrated that the combinatorial model (blue bars) could provide the overall best performance, with balanced sensitivity and specificity, in the prediction of carbonylation sites.

### Construction of web-based prediction tool

**Fig. 5:**
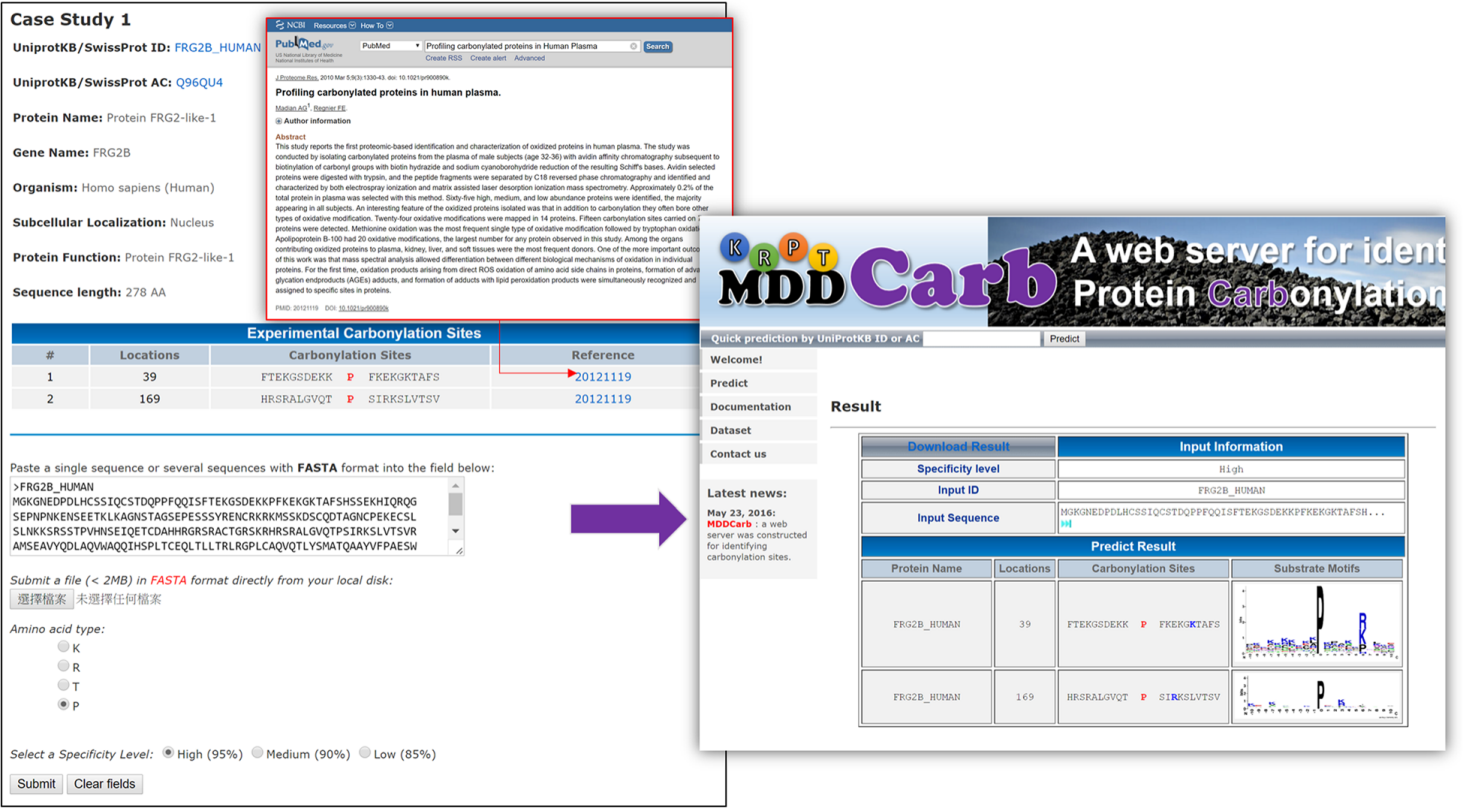
A case study of carbonylation sites prediction on Protein FRG2-like-1 (FRG2B)

Because the experimental identification of site-specific carbonylated peptides is labor-intensive, many tools have been developed for the computational prediction of carbonylation sites. However, there exists no method dedicated to the characterization of potential substrate motifs of carbonylated sites. Thus, we were inspired to develop a user-friendly web tool, named MDD-Carb, for identifying the carbonylation sites with corresponding substrate motifs. The combinatorial model, integrating the MDDLogo-identified motif signatures, was adopted to implement the prediction function on the website. Users are allowed to submit their protein sequences in FASTA format, and the prediction function returns the results, including carbonylated positions as well as the flanking amino acids. Additionally, the substrate motifs corresponding to the predicted carbonylation sites are also available. As a case study shown in Fig. 5, Protein FRG2-like-1 (FRG2B) has two confirmed carbonylation sites, P39 and P169 [59]. After the submission of a whole protein sequence, the MDD-Carb could effectively identify the two carbonylated sites with their corresponding motifs. The MDD-Carb is anticipated to facilitate the study of large-scale carbonylated proteomes, and it is now freely available to all interested users at http://csb.cse.yzu.edu.tw/MDDCarb/.

## Conclusion

In this work, we investigated the amino acid composition near verified carbonylation sites systematically. This investigation showed that the occurrence rates of K, R, T, and P were higher in the carbonylation sites than those in non-carbonylation sites, in which K is significantly abundant. Based on the five-fold cross-validation, the SVM models trained with AAC or with PWM provided the best performance out of the SVM models in identifying carbonylation sites. After the application of MDDLogo on positive training datasets, the profile HMMs trained from MDDLogo-clustered subgroups, which contained statistically significant motif signatures, presented an enhanced performance compared to that of the models without MDD clustering. To conduct a reasonable integration of multiple profile HMMs, a combinatorial model was developed by incorporating multiple profile HMMs into a single predictive model. The independent testing results demonstrated that the combinatorial model provided the overall best predictive performance with balanced sensitivity and specificity.

## Additional files

Additional file 1: Table S1. Summary list of previously published methods for predicting protein carbonylation sites (DOCX 19 kb)

Additional file 2: Figure S1. System flow of the combinatorial model incorporating SVM with profile HMMs (DOCX 229 kb)

Additional file 3 Figure S2. Frequency plots of carbonylated K, R, T, and P residues by using WebLogo (DOCX 675 kb)

Additional file 4: Figure S3. Comparison of ROC curves between the profile HMM and SVM models trained with various features for the identification of carbonylated K sites based on five-fold cross-validation (DOCX 199 kb)

Additional file 5: Figure S4. Comparison of ROC curves between the profile HMM and SVM models trained with various features for the identification of carbonylated R sites based on five-fold cross-validation (DOCX 202 kb)

Additional file 6: Figure S5. Comparison of ROC curves between the profile HMM and SVM models trained with various features for the identification of carbonylated T sites based on five-fold cross-validation (DOCX 222 kb)

Additional file 7: Figure S6. Comparison of ROC curves between the profile HMM and SVM models trained with various features for the identification of carbonylated P sites based on five-fold cross-validation (DOCX 198 kb)

Additional file 8: Table S2. The performance of profile HMMs trained with MDDLogo-identified substrate motifs based on five-fold cross-validation (DOCX 323 kb)
