# Supplementary material for: MDD-carb: a combinatorial model for the identification of protein carbonylation sites with substrate motifs
Source: BMC Syst Biol. 2017 Dec 21;11(Suppl 7):137. doi: 10.1186/s12918-017-0511-4 (PMC5763492; doi:10.1186/s12918-017-0511-4)
Supplement: Supplementary file 1 — Summary list of previously published methods for predicting protein carbonylation sites (DOCX 19 kb) [file 12918_2017_511_MOESM1_ESM.docx]

**Table S1. Summary list of previously published methods for predicting protein carbonylation sites.**

| Method | Window  size | Positive /Negative ratio | Method | Features | Species | Cross-validation | A.A. | Performance  (Accuracy) |
| --- | --- | --- | --- | --- | --- | --- | --- | --- |
| **CarSPred**  (Lv, H. et al., 2014) | 27 | 1:6 | SVM | PSPAKSAP | Mammals | 10-fold | K | 85.72% |
|  |  |  |  |  |  |  | P | 85.72% |
|  |  |  |  |  |  |  | R | 85.95% |
|  |  |  |  |  |  |  | T | 83.92% |
| **iCar-PseCp**  (Jia, J. et al., 2016) | 15 | 1:6 | Random forest | PseAAC | Human Photobacterium Escherichia coli | 10-fold | K | 84.43% |
|  |  |  |  |  |  |  | P | 86.79% |
|  |  |  |  |  |  |  | R | 84.23% |
|  |  |  |  |  |  |  | T | 86.17% |
| **predCar-site**  (Hasan, M.A., et al., 2017) | 15 | 1:6 | SVM | PseAAC | Photobacterium  Escherichia coli | 10-fold | K | 96.95% |
|  |  |  |  |  |  |  | P | 99.61% |
|  |  |  |  |  |  |  | R | 99.10% |
|  |  |  |  |  |  |  | T | 99.11% |
| **CarbSite**  (Weng, S.L. et al., 2017) | 21 | 1:5 | SVM + Random forest | AAC + PWM + AAindex | Mammals | 5-fold | K | 77.10% |
|  |  |  |  |  |  |  | P | 77.90% |
|  |  |  |  |  |  |  | R | 79.50% |
|  |  |  |  |  |  |  | T | 78.80% |
