# Supplementary material for: MDD-carb: a combinatorial model for the identification of protein carbonylation sites with substrate motifs
Source: BMC Syst Biol. 2017 Dec 21;11(Suppl 7):137. doi: 10.1186/s12918-017-0511-4 (PMC5763492; doi:10.1186/s12918-017-0511-4)
Supplement: Supplementary file 2 — System flow of the combinatorial model incorporating SVM with profile HMMs (DOCX 229 kb) [file 12918_2017_511_MOESM2_ESM.docx]

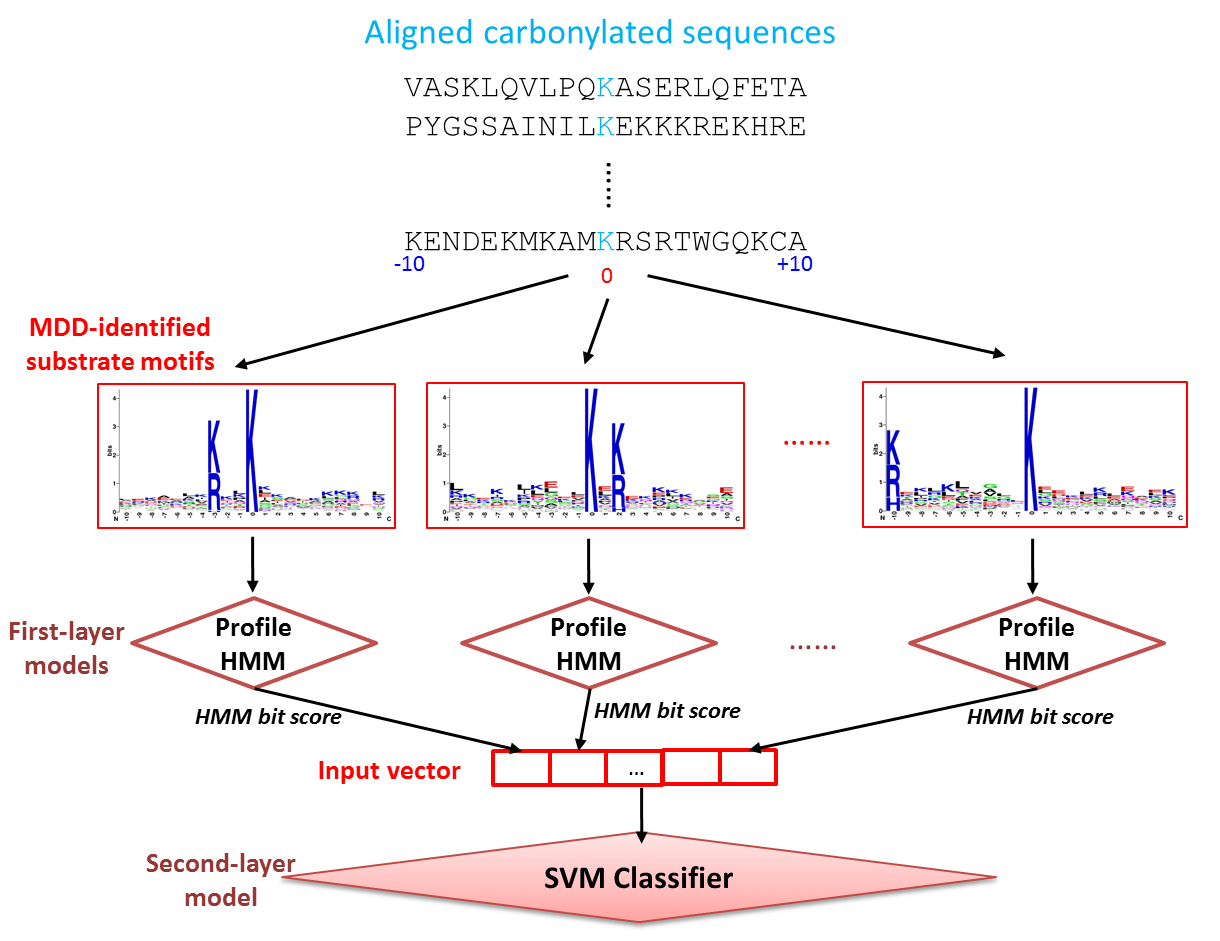


**Figure S1. System flow of the combinatorial model incorporating multiple profile HMMs into a single SVM model.**
