# Supplementary figures and images for: MDD-carb: a combinatorial model for the identification of protein carbonylation sites with substrate motifs
Source: BMC Syst Biol. 2017 Dec 21;11(Suppl 7):137. doi: 10.1186/s12918-017-0511-4 (PMC5763492; doi:10.1186/s12918-017-0511-4)

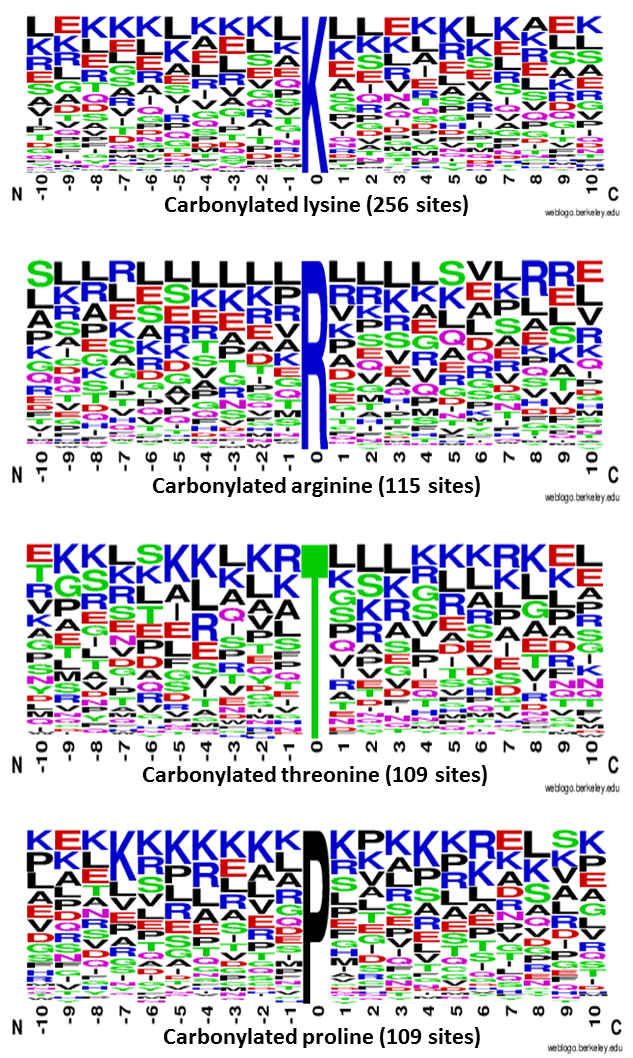


**Figure S2. Frequency plots of carbonylated K, R, T, and P residues by using WebLogo.**

Supplement: Supplementary file 3 — Frequency plots of carbonylated K, R, T, and P residues by using WebLogo (DOCX 675 kb) [file 12918_2017_511_MOESM3_ESM.docx]
