# Supplementary material for: MDD-carb: a combinatorial model for the identification of protein carbonylation sites with substrate motifs
Source: BMC Syst Biol. 2017 Dec 21;11(Suppl 7):137. doi: 10.1186/s12918-017-0511-4 (PMC5763492; doi:10.1186/s12918-017-0511-4)
Supplement: Supplementary file 6 — Comparison of ROC curves between the profile HMM and SVM models trained with various features for the identification of carbonylated T sites based on five-fold cross-validation (DOCX 222 kb) [file 12918_2017_511_MOESM6_ESM.docx]

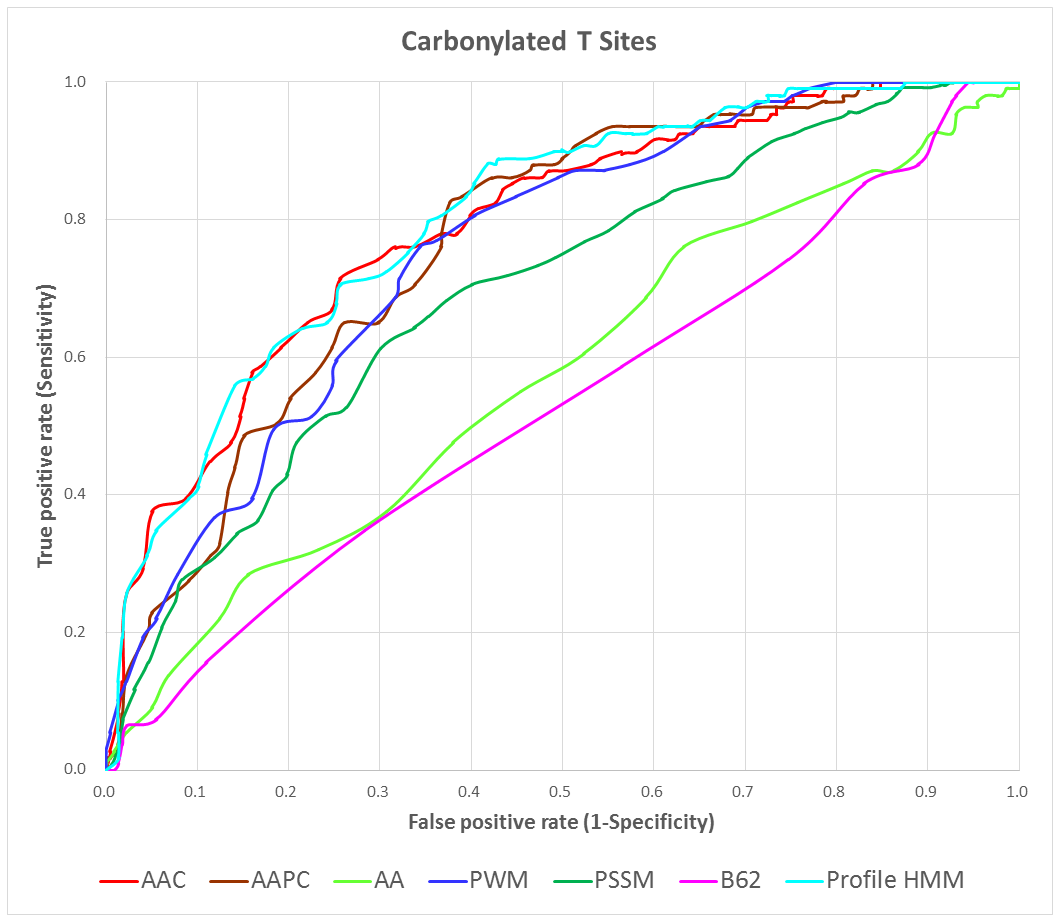


**Figure S5. Comparison of ROC curves between profile HMM and the SVM models trained with various features for the identification of carbonylated T sites based on five-fold cross-validation.**
