# Supplementary material for: MDD-carb: a combinatorial model for the identification of protein carbonylation sites with substrate motifs
Source: BMC Syst Biol. 2017 Dec 21;11(Suppl 7):137. doi: 10.1186/s12918-017-0511-4 (PMC5763492; doi:10.1186/s12918-017-0511-4)
Supplement: Supplementary file 8 — The performance of profile HMMs trained with MDDLogo-identified substrate motifs based on five-fold cross-validation (DOCX 323 kb) [file 12918_2017_511_MOESM8_ESM.docx]

Table S2. The performance of profile HMMs trained with MDDLogo-identified substrate motifs based on five-fold cross-validation

| **MDDLogo-clustered subgroup** | **Entropy plot** | **Number of positive data** | **Number of negative data** | **Sn** | **Sp** | **Acc** | **MCC** | **AUC** |
| --- | --- | --- | --- | --- | --- | --- | --- | --- |
| All carbonylated K sites | 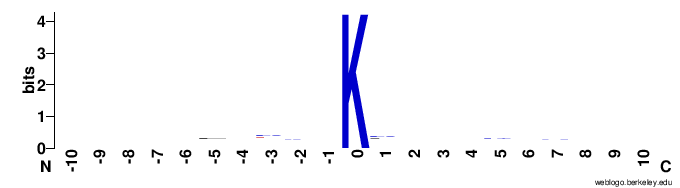 | 256 | 512 | 0.72 | 0.71 | 0.71 | 0.41 | 0.81 |
| CarbK_1 | 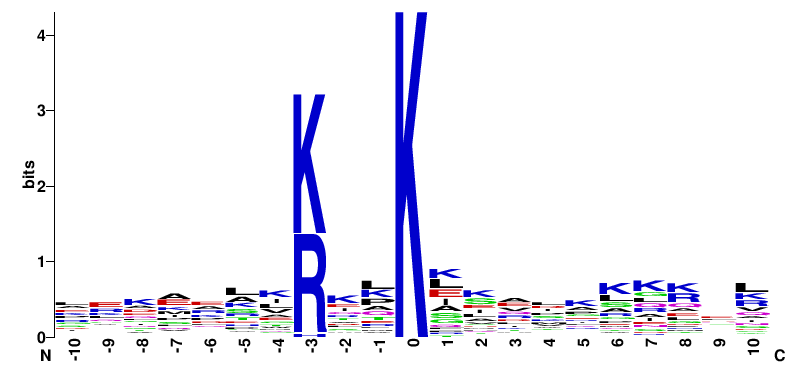 | 54 | 108 | 0.81 | 0.81 | 0.81 | 0.61 | 0.91 |
| CarbK_2 | 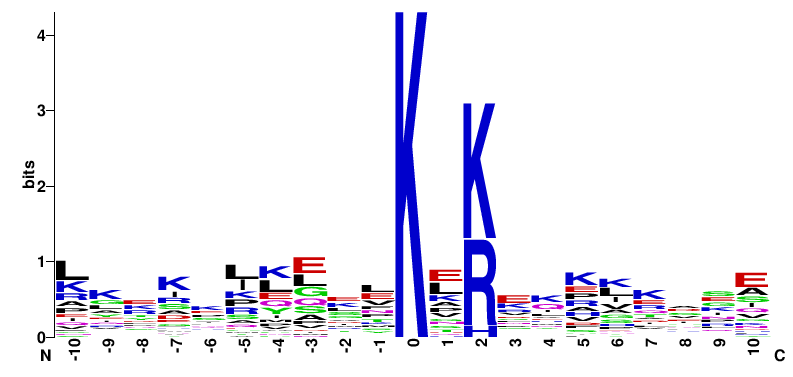 | 52 | 104 | 0.74 | 0.74 | 0.74 | 0.46 | 0.84 |
| CarbK_3 | 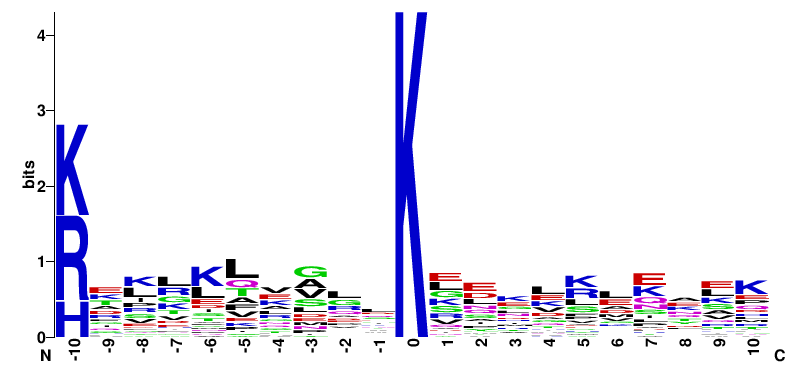 | 35 | 70 | 0.71 | 0.71 | 0.71 | 0.41 | 0.81 |
| CarbK_4 | 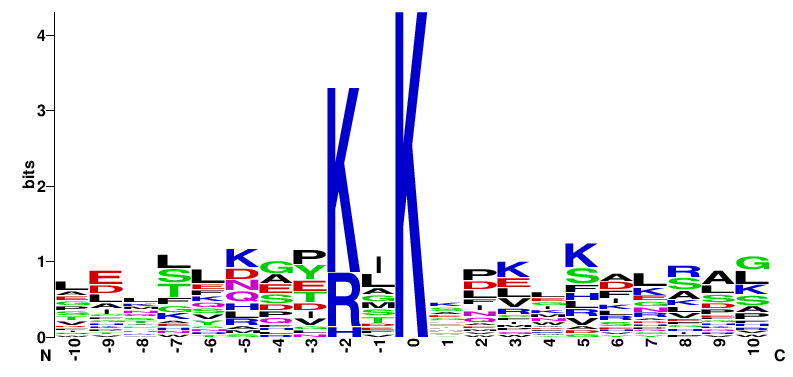 | 33 | 66 | 0.77 | 0.82 | 0.80 | 0.59 | 0.89 |
| CarbK_5 | 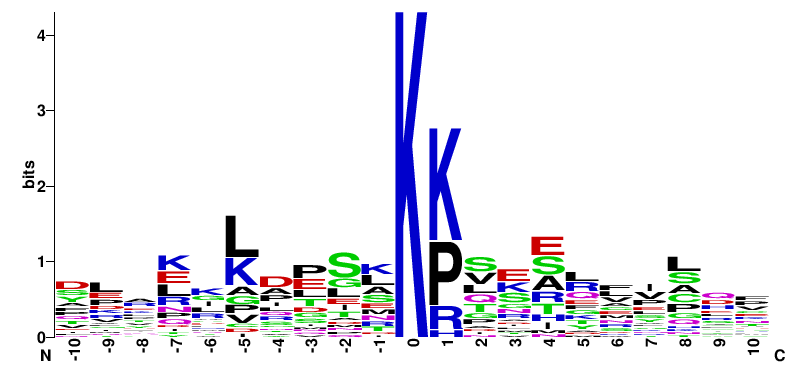 | 26 | 52 | 0.69 | 0.75 | 0.73 | 0.43 | 0.82 |
| CarbK_6 | 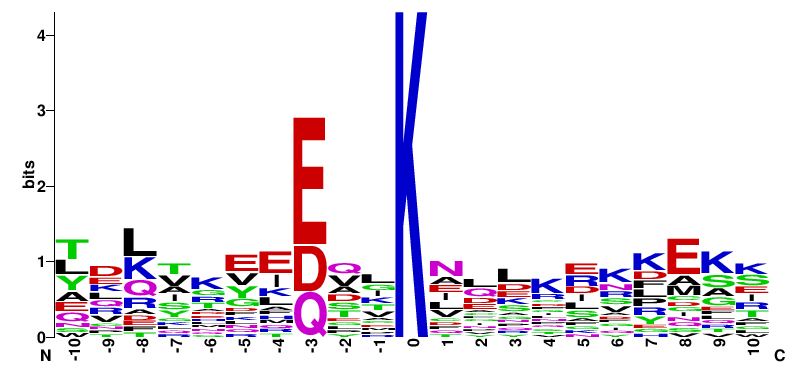 | 33 | 66 | 0.76 | 0.76 | 0.76 | 0.49 | 0.85 |
| CarbK_7 | 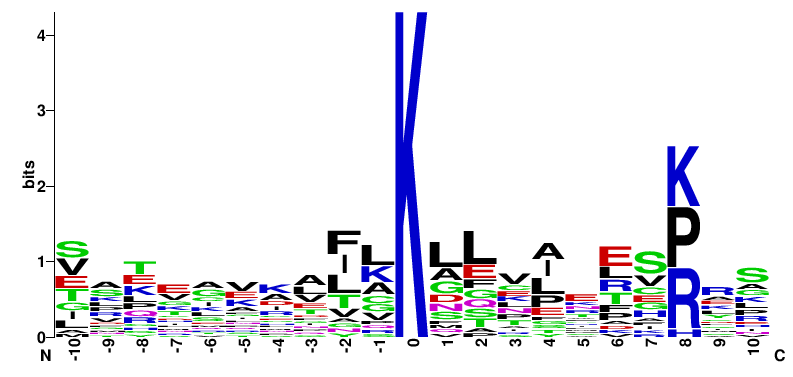 | 23 | 46 | 0.74 | 0.74 | 0.74 | 0.45 | 0.84 |
| All carbonylated R sites | 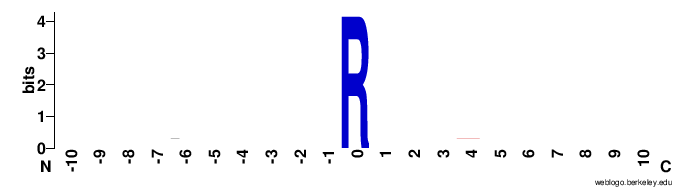 | 115 | 230 | 0.68 | 0.66 | 0.67 | 0.33 | 0.73 |
| CarbR_1 | 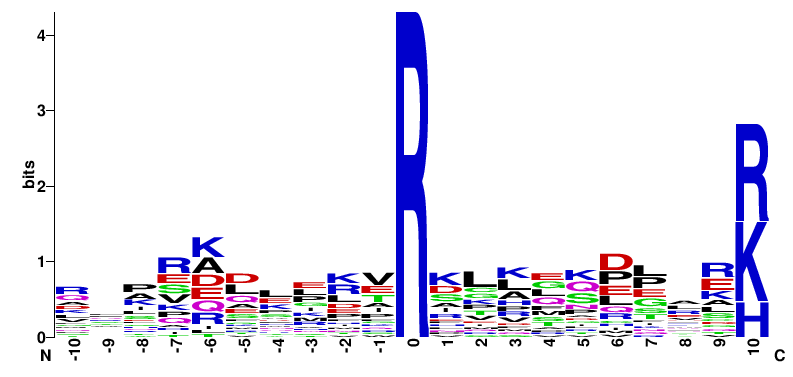 | 56 | 92 | 0.76 | 0.74 | 0.75 | 0.47 | 0.85 |
| CarbR_2 | 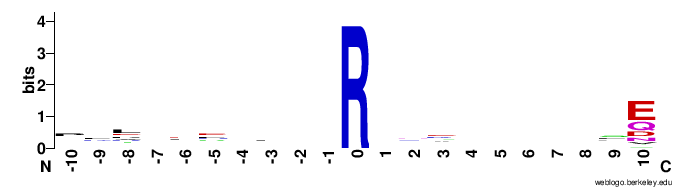 | 33 | 86 | 0.70 | 0.68 | 0.69 | 0.35 | 0.75 |
| All carbonylated T sites | 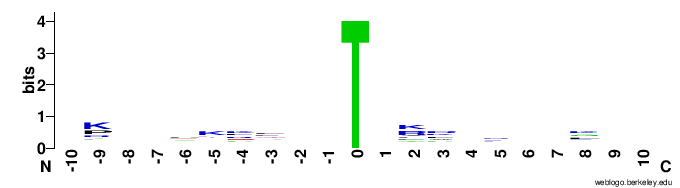 | 109 | 218 | 0.70 | 0.69 | 0.69 | 0.37 | 0.77 |
| CarbT_1 | 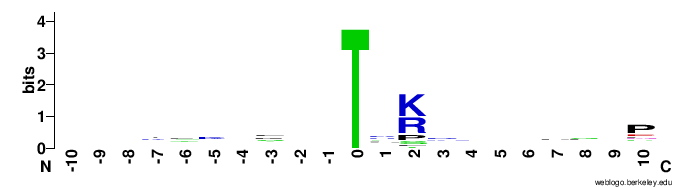 | 46 | 92 | 0.74 | 0.74 | 0.74 | 0.45 | 0.84 |
| CarbT_2 | 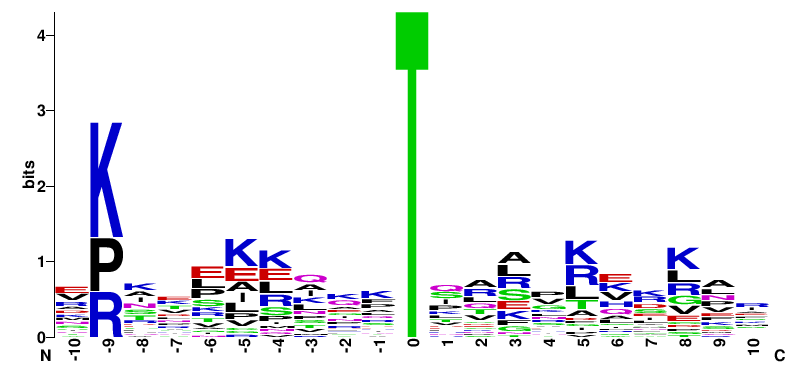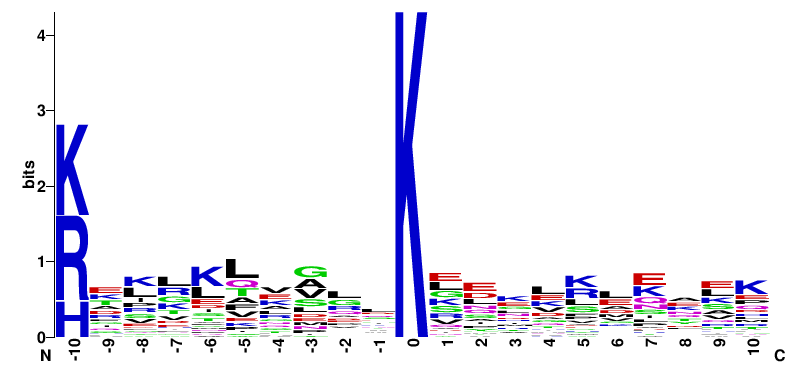 | 38 | 76 | 0.75 | 0.75 | 0.75 | 0.48 | 0.85 |
| All carbonylated P sites | 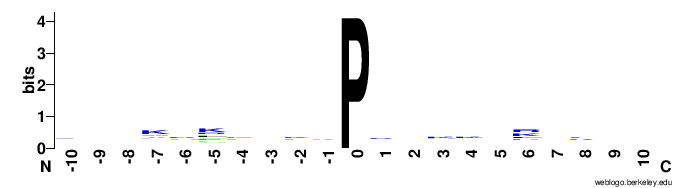 | 109 | 218 | 0.72 | 0.72 | 0.72 | 0.42 | 0.82 |
| CarbP_1 | 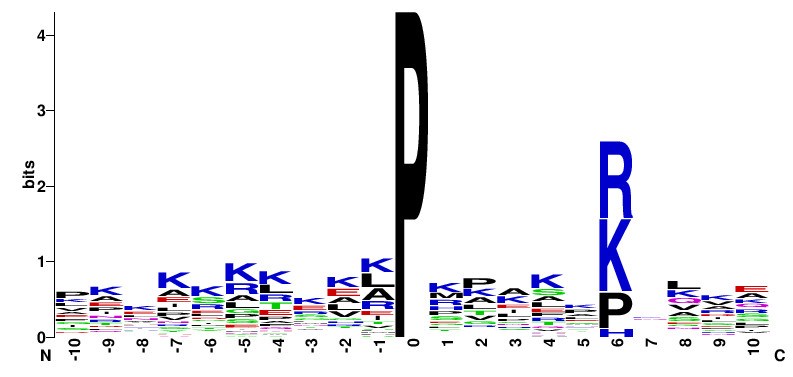 | 43 | 86 | 0.81 | 0.81 | 0.81 | 0.61 | 0.90 |
| CarbP_2 | 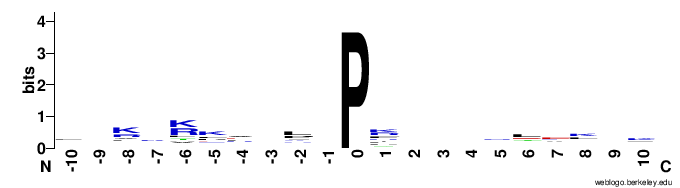 | 22 | 44 | 0.73 | 0.73 | 0.73 | 0.43 | 0.82 |
| CarbP_3 | 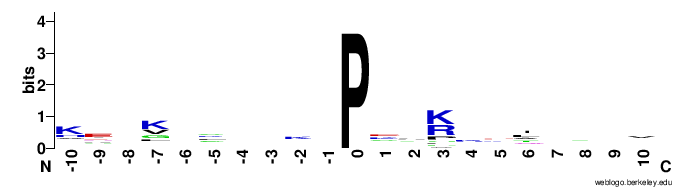 | 21 | 42 | 0.70 | 0.68 | 0.69 | 0.34 | 0.73 |
